# The Red Fox (*Vulpes vulpes*) as Sentinel for Tick-Borne Encephalitis Virus in Endemic and Non-Endemic Areas

**DOI:** 10.3390/microorganisms8111817

**Published:** 2020-11-18

**Authors:** Maja Haut, Philipp Girl, Beate Oswald, Thomas Romig, Anna Obiegala, Gerhard Dobler, Martin Pfeffer

**Affiliations:** 1 Institute of Animal Hygiene and Veterinary Public Health, Faculty of Veterinary Medicine, University of Leipzig, 04103 Leipzig, Germany; Maja.Haut@vetmed.uni-leipzig.de (M.H.); Anna.Obiegala@vetmed.uni-leipzig.de (A.O.); 2 German National Consultant Laboratory for TBEV, Bundeswehr Institute of Microbiology, 80937 Munich, Germany; Philipp1Girl@bundeswehr.org (P.G.); BeateOswald@bundeswehr.org (B.O.); gerharddobler@bundeswehr.org (G.D.); 3 Parasitology Unit, Institute of Zoology, University of Hohenheim, 70599 Stuttgart, Germany; Thomas.Romig@uni-hohenheim.de

**Keywords:** Flavivirus, ELISA, IIFA, micro-NT, seroprevalence, TBE, Europe

## Abstract

Tick-borne encephalitis (TBE) is one of the most important viral zoonosis caused by a neurotropic arbovirus (TBEV). In Germany, TBE is classified as a notifiable disease with an average of 350 autochthonous human cases annually. The incidence-based risk assessment in Germany came under criticism because every year, a number of autochthonous human TBE cases have been detected outside of the official risk areas. Therefore, it is necessary to find additional parameters to strengthen TBEV surveillance. The aim of this study was to examine red foxes as sentinels for TBE. Thus far, there are no published data about the sensitivity and specificity for serological methods testing fox samples. Hence, we aimed to define a system for the screening of TBEV-specific antibodies in red foxes. A total of 1233 fox sera were collected and examined by ELISA and IIFA and confirmed by micro-NT. The overall seroprevalence of antibodies against TBEV in red foxes from Germany confirmed by micro-NT was 21.1%. The seroprevalence differed significantly between risk (30.5%) and non-risk areas (13.1%), with good correlations to local TBE incidence in humans. In conclusion, serological monitoring of red foxes represents a promising surrogate marker system and may even determine unexpected TBEV foci in regions currently regarded as non-risk areas.

## 1. Introduction

Tick-borne encephalitis (TBE) is a medically relevant viral zoonosis transmitted through bites of certain tick species. TBE is caused by the tick-borne encephalitis virus (TBEV), which belongs to the genus Flavivirus, family Flaviviridae. According to Ecker et al. (1999), TBEV can be divided into three subtypes: European, Siberian and Far Eastern subtype [1]. However, recent findings suggest a division of TBEV in at least five genetic subtypes. In addition to the three named subtypes, a Baikalian [2] and a Himalayan subtype [3] have been described. The Siberian and Far Eastern subtypes are mainly transmitted by *Ixodes persulcatus*, while the European subtype mainly uses *Ixodes ricinus* as a vector.

All subtypes of TBEV circulate between small mammals as main reservoir hosts, other mammalian hosts, and ixodid ticks as vectors in geographically limited natural foci [4]. The concept of the natural focus was initially formulated by Pavlovsky shortly after TBEV was isolated [5]. According to our current understanding, a natural focus consists of one small area of 0.005 km^2^ (‘microfocus’) located within a larger area (‘macrofocus’), which extends 1 km around the microfocus [6,7]. In the microfocus, an active continuous transmission of TBEV between ticks and rodents takes place while rodents or other larger animals like foxes, wild boars, or deer transport the infected ticks into the macrofocus [6,8]. Due to the synanthropic behavior of many wild animals, such as red foxes, the risk of dispersal of TBEV-infected ticks into villages and towns increases [6,9].

Currently, TBEV foci have been reported in Europe, Siberia, far-eastern Russia, northern China, South Korea, and Japan, with approximately 10,000–12,000 human cases per year [10]. Since the introduction of mandatory reporting in Germany in 2001, an average of 350 autochthonous human cases have been notified annually [11]. In Germany, TBE risk areas are defined on a district level by a minimal incidence of 1 case per 100,000 inhabitants in a gliding five-year period [12]. Besides the different geographical scale, in comparison with the natural focus, further drawbacks of this definition are that they disregard the vaccination rates and geographical inaccuracies, as notification is done by the place of living of the patient rather than the place of infection. Furthermore, humans are only incidental hosts and up to 70% of human infections are estimated to manifest without or with only mild clinical symptoms, which usually does not lead to any diagnostic examinations. In consequence, increasing vaccination rates may reduce the number of clinical TBE cases and thus the number of official TBE risk areas, although there is a continuous circulation of TBEV in the natural foci and thus a continuous risk of human infections. Additionally, in Germany, every year around 3% of the autochthonous human TBE cases are detected outside of the official risk areas [11]. Therefore, it is necessary to find suitable alternative parameters for TBEV surveillance to complement or modify the existing risk assessment based solely on the number of human cases [13,14].

In epidemiological studies, testing of wild animals or domestic animals by serological means has proven to be the best method for the evidence of TBEV infections [15]. For this purpose, different serological test formats are used. The most common screening methods are the indirect immunofluorescence assay (IIFA) and the enzyme-linked immunosorbent assay (ELISA). The difficulty here is that both screening systems show cross-reactivity to antibodies of other flaviviruses [16]. Hence, it is necessary to distinguish between specific anti-TBEV antibodies and antibodies against other flaviviruses by using, e.g., an additional neutralization test (NT). According to the European Centre for Disease Prevention and Control, only an NT titer ≥1:10 confirms a positive result [17].

Many wild and domestic animals (e.g., roe deer, wild boar, dogs, goats, sheep) are hosts for ticks and can develop measurable antibody titers against TBEV upon natural infection and were previously investigated as potential new indicators of TBE infection risk [18,19,20]. Although red foxes are possible transporters of *Ixodes* spp. ticks into human habitat [21], and are predators of potentially TBEV-infected rodents [22], there are no recent studies investigating the TBEV seroprevalence in foxes. In previous studies from Germany and the Netherlands, only one serum sample was confirmed by a neutralization test. The remaining fox sera were only tested by ELISA or were negative in NT [14,23,24]. However, Swedish researchers found a positive correlation between TBE incidence and fox abundance [21], while a study from Germany described the opposite effect [25].

These contradictory findings clearly demonstrate the necessity of a seroprevalence study in areas with known endemicity status in order to see whether or not foxes develop antibodies upon infection, which might be useful as a surrogate marker for TBE endemic areas. Thus far, there are no studies calculating the sensitivity and specificity of commercial serological test systems for foxes. Therefore, an additional aim of this study was the comparison of serological methods to select the best for TBEV antibody detection in foxes. Subsequently, we determined the seroprevalence of antibodies against TBEV in red foxes from Germany in risk areas and non-risk areas and correlated TBE incidence in humans and fox seroprevalence in order to estimate the potential of red foxes as possible sentinels of human TBE risk.

## 2. Materials and Methods

### 2.1. Animal Collection and Serum Samples

Between December 2016 and February 2020, we collected foxes from local hunters shot during traditional hunting. This was done in cooperation with a scrape station (Fellwechsel GmbH, Berlin, Germany) and an echinococcosis project of the Parasitology Unit of the University of Hohenheim. Local hunters from all over Germany deposited fox carcasses in plastic bags labelled with the date and exact geographical location in deep freezers. Only adult foxes with good nutritional status were selected by hunters for ‘Fellwechsel’, while juvenile foxes were also shot for the project of the university. Fox cadavers were stored at −80 °C (Fellwechsel) or −20 °C (University) until flaying (Fellwechsel) or parasitological diagnostic examination (University).

For the extraction of thoracic fluid, 5 mL of Phosphate-buffered saline (PBS) were flushed into the thorax of fox carcasses. Alternatively, if the thorax was destroyed, the heart of the foxes was punctured and flushed with 5 mL of PBS. The flushing liquid of both was centrifuged (10 min at 2000× *g*) and stored at −20 °C, further considered as serum samples.

### 2.2. Enzyme-Linked Immunosorbent Assay (ELISA)

All serum samples were tested using the Immunozym FSME IgG All Species ELISA (Progen, Heidelberg, Germany) according to the manufacturer’s instructions. Serum samples were tested in a dilution of 1:50 and measured in Vienna Units per milliliter (VIEU/mL). An ELISA reader (Thermo Fisher Scientific Inc., Waltham, MA, USA) was used for the colorimetric reading at the 450-nm wavelength. Sera with concentration >126 VIEU/mL were considered as positive, sera from 63–126 VIEU/mL as borderline, and concentrations <63 VIEU/mL were considered as negative.

### 2.3. Indirect Immunofluorescence Assay (IIFA)

All positive and borderline sera were also tested in an indirect immunofluorescence assay (IIFA). Additionally, 71 randomly chosen fox sera from TBE endemic areas, which were tested negative in ELISA, were also tested in IIFA. For the IIFA analysis, the Euroimmun Anti-TBEV-IIFT (FSME-Viren [TBEV], Euroimmun AG, Luebeck, Germany) was used. Serum samples were used with a dilution of 1:10 with PBS. A 20-µL aliquot of each sample was spotted on a field of the TBE microchip and incubated for 30 min at 37 °C in a humidified atmosphere. After washing 3 times in PBS, 10 µL of anti-dog FITC conjugate were added and again incubated for 30 min at 37 °C in a humidified incubator. After 3 washing steps in PBS, the slides were mounted with 10% glycerol in PBS and checked for specific immunofluorescence. In each field, an infected field was opposite to a field with non-infected cells as a control for unspecific fluorescence.

### 2.4. Micro-Neutralization Test (Micro-NT)

In order to avoid false positive results, all ELISA- and IIFA-positive sera were confirmed by micro-NT as the ECDC requires NT titers of ≥1:10 to be regarded as confirmed positive for TBE-specific antibodies. Additionally, all negative samples were re-tested for the purpose of determining the sensitivity and specificity of ELISA and IIFA.

Micro-NT analysis of serum samples was performed according to standard procedure as described before [26]. In brief, virus stocks (50 TCID/50 µL) were prepared from TBE (strain Neudörfl) cultivated in A549 cells and stored at −80 °C until further use. Using 96-well culture plates (Greiner bio-one, Frickenhausen, Germany), all micro-NTs were performed on confluent cell monolayers. Serum samples were tested in duplicates, diluted in Minimal Essential Medium (MEM, plus Non-Essential Amino Acids Solution and Antibiotic-Antimycotic Solution; all Invitrogen, ThermoFisher Scientific, Darmstadt, Germany) in a ratio of 1:10. As a control, a known positive and a known negative serum sample were used on each plate together with a mock control and a virus back-titration. Diluted serum samples were pre-incubated with virus for one hour before adding the serum virus suspension to the wells. Supernatants were discarded after an incubation period of 96 h at 37 °C (5% CO_2_), and the 96-well plates were fixed and stained in 13% formalin/0.1% crystal violet and results determined optically. Samples were classified as either “micro-NT-positive” (titer ≥ 1:10) if there was complete inhibition of the cytopathic effect in both wells of the cell culture or “micro-NT-negative” (titer < 1:10) otherwise.

### 2.5. Statistical Analysis

The independence of compared sample sizes over 50 was tested with Chi-square test. Correlations between the seroprevalence of TBE-specific antibodies in red foxes in risk and non-risk areas and the local TBE incidence in humans were evaluated using Pearson’s correlation coefficient and Spearman’s rank correlation coefficient. Statistical analyses were performed using IBM SPSS Statistics for Windows, v. 25.0 (IBM Corp, Armonk, NY, USA).

## 3. Results

### 3.1. Animal Collection

In total 1233 red fox serum samples were collected. Of these, 59.0% (*n* = 728/1233) were from male foxes, 40.2% (*n* = 496/1233) from female foxes, and in 0.7% (*n* = 9/1233), the gender could not be determined due to extensive damage of the lower abdomen. Most of the foxes were adult (98.5%; *n* = 1214/1233) and only 1.5% (*n* = 19/1233) were juvenile. For the present study, sera were collected from districts all over Germany. To enhance the accuracy of our analyses, these districts were divided into TBE risk (*n* = 568/1233; 46.1%) (Table 1) and TBE non-risk areas (*n* = 665/1233; 53.9%) according to the Robert Koch-Institute [27] (Table 2).

### 3.2. ELISA

On the whole, 121/1233 samples were positive or borderline by TBE ELISA (Table 3). Among these sera, 110 samples could be confirmed by micro-NT. In total, 150 additional sera were positive in the micro-NT, which yielded negative results in the ELISA. In total, 973 samples were confirmed to be negative for TBEV-specific antibodies by micro-NT. Of these, 11 samples tested positive by ELISA. Therefore, the sensitivity of the Immunozym FSME IgG All Species ELISA (Progen, Heidelberg, Germany) was 42.3% in classifying all positive and borderline ELISA samples as positive. The specificity of the Immunozym FSME IgG All Species ELISA was 98.9%.

### 3.3. IIFA

In total, 192 samples were tested by IIFA. Of these, 138 samples were considered positive. However, 26 samples, which showed a positive reaction in IIFA, were negative in micro-NT and thus not confirmed. On the other hand, out of the 54 samples that tested negative by IIFA, 11 were tested positive in the micro-NT. As the results of ELISA and micro-NT corresponded better, a further testing of samples using IIFA was discontinued because an even lower specificity and sensitivity was expected for this screening method.

### 3.4. Micro-NT

**Figure 1:**
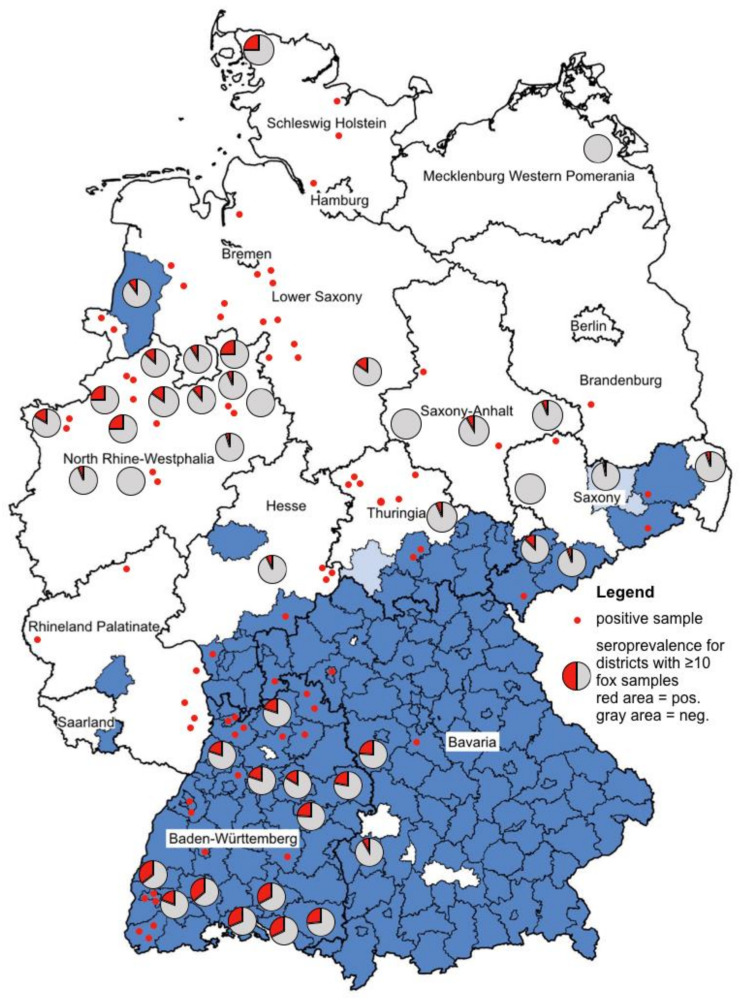
Blue colored are the TBE risk areas (counties, NUTS 3 level) according to Robert Koch Institute [27]. The lighter blue are the districts that were newly accounted for TBE risk areas in 2020. Pie charts were created for the districts where 10 or more foxes were examined. The red areas of the pie charts stand for the positive foxes, the gray ones for the negatively tested foxes. Positive foxes from districts where less than 10 foxes were sampled are represented by a red dot. Please note that negative foxes (*n* = 315) from districts where less than 10 foxes were sampled are not shown in this figure.

By using only sera confirmed by micro-NT, the overall seroprevalence of antibodies against TBEV in foxes from Germany was 21.1% (*n* = 260/1233). The prevalence of TBEV-specific antibodies in male and female foxes did not differ significantly (χ^2^ = 0.241, df = 1, *p* = 0.6238). In TBE risk areas, the seroprevalence was 30.5% (*n* = 173/568), and in non-risk areas only 13.1% (*n* = 87/665). Significantly more foxes from TBE risk areas developed TBEV-specific antibodies in comparison with foxes from non-risk areas (χ^2^ = 35.184, df = 1, *p* < 0.001). For calculation of this correlation, only districts were selected where 10 or more foxes were sampled. The TBEV seroprevalence in foxes from risk areas correlated significantly with the human TBE incidence in these areas (*r_s_* = 0.608, *p* = 0.006, *n* = 19; *r* = 0.608, *p* = 0.006, *n* = 19), but the correlation in non-risk areas was not significant (*r_s_* = −0.346, *p* = 0.1006, *n* = 23; *r* = −0.282, *p* = 0.192, *n* = 23). The geographical distribution of foxes with TBEV-specific antibodies are shown in Figure 1.

## 4. Discussion

An important aim of our study was to define a system that allows the screening of TBEV-specific antibodies in red foxes. Thus far, no published data existed about the sensitivity and specificity of commercially available screening methods for the detection of antibodies against TBEV in sera from red foxes. Therefore, a commercially available ELISA kit (Immunozym FSME IgG All Species ELISA) and a commercially available IIFA assay (Euroimmun Anti-TBEV-IIFT) were compared using the NT as the “gold standard”. However, after testing 192 samples, further use of IIFA was discontinued because the specificity of IIFA was even lower than that of the ELISA (Table 4 and Table 5). The sensitivity of both test systems at this intermediate stage is comparably high, as mainly positive samples were tested.

After all, 1233 samples were tested by ELISA and NT, the ELISA showed a sensitivity of 42.3% and specificity of 98.9% (Table 3). Klaus et al. (2011) detected similar levels of sensitivity and specificity using the Immunozym FSME IgG All Species ELISA for goat serum samples [28]. Apparently, this ELISA is a good method for diagnosis of acute TBEV infection, but positive serum samples may be missed if the antibody titer is low after a long-lasting natural infection or when using diluted serum samples (manuscript in preparation). Hence, a two-step diagnostic system with a screening ELISA or IIFA and a confirmatory NT might not be suitable for epidemiological investigations. The question therefore arises whether for epidemiological investigations, for example, the search for a suitable sentinel, only an NT should be performed.

The neutralization test is the most specific and sensitive assay, which exploits the capacity of antibodies to neutralize infectious viruses [16,28]. Several different formats are available, for example, it is possible to perform a plaque reduction neutralization test, assessing the serum dilution at which the number of viral plaque-forming units is reduced by 50% or 90% [29], or to assess the tissue culture infectious dose by the microneutralization test [16]. However, the major disadvantages of NT are the need of a BSL3 laboratory for TBE culture, the requirement of highly trained staff, and high time consumption [30]. We designed a protocol for a microneutralization test, which was done with only one dilution and in duplicate. The advantage of this test format is that up to 40 sera can be tested in one 96-well plate and therefore this test may be suitable for mass testing in epidemiological studies. As for epidemiological studies, time is not a critical factor, and this disadvantage of NT for acute diagnosis might not be relevant. Additionally, one advantage of NT is that it works species independently. Therefore, the presented micro-method can be recommended for epidemiological investigation of any species of wild animals.

A further difficulty is the poor quality of serum samples from fox carcasses and other wild animals [13,18]. Due to extensive hemolysis, contamination, or toxic effects, the evaluation by NT is sometimes impossible [18]. We prepared our samples by centrifugation and inactivation at 56 °C to eliminate possible contaminations, other pathogens and the complement factors [31,32].

The second major aim of the study was to explore the potential of red foxes as predictive sentinels for TBE risk. In accordance with Gerth et al. (1995), the ideal sentinel should meet four conditions: “have an adequately limited home range, be available in large numbers, be well dispersed in the surveillance area, and should show a long-lasting antibody response after natural infection” [33]. The use of the home range of foxes depends on the availability of resources and habitat, hence the home range size of rural foxes is 2 km^2^ and of urban foxes 0.5 km^2^ [9]. Furthermore, the high adaptability of foxes, their omnivorous diet, and the absence of natural enemies contribute to a broad distribution in Europe [34,35,36]. The duration of antibody response in foxes is still unknown, but after experimental infection via infected ticks or virus injection, foxes show clinical symptoms like fever and meningoencephalitis, a viremia, and high antibody titers [37,38].

In addition, the ideal sentinel should be exposed frequently to tick species known as vectors of TBEV [13]. In Europe, tick infestation of foxes ranges from 7.4% in Italy [39] to 51.1–82.6% in Spain [40] and Germany [41]. Several tick species of the genera Ixodes, Dermacentor, Haemaphysalis, Hyalomma, and Rhipicephalus are known to parasitize red foxes’ skin [39,40,42,43]. Of these, three species, *I. ricinus*, *D. reticulatus*, and *H. concinna*, are known to be able to transmit TBEV. Moreover, the virus has been isolated from *I. hexagonus*, *I. canisuga*, *H. punctata*, and *I. trianguliceps*, typical members of the tick fauna of red foxes in Europe [44]. In Croatia, TBEV has been isolated from *I. ricinus* and *I. hexagonus* ticks removed from red foxes [45].

In conclusion, the red fox meets all the requirements to be a suitable indicator for TBE risk assessment, which is also confirmed by our results. The observed seroprevalence of TBEV antibodies reflects the TBE infection rate in Germany, with significant differences between risk (*n* = 173/568; 30.5%) and non-risk areas (*n* = 87/665; 13.1%). At least 87 TBEV-positive fox serum samples were from districts officially classified as non-risk areas. These include one district in Brandenburg; two districts in Hesse and Saxony; four districts each in Rhineland-Palatinate, Saxony-Anhalt, and Schleswig-Holstein; five districts in Thuringia; 10 in Lower Saxony, and 16 in North-Rhine-Westphalia. Although most of these districts are only represented by a small sample size, these examples demonstrate the tremendous power of using foxes to detect new areas of TBEV activity. In addition, as all samples become diluted by the flushing fluid during sampling, an even higher seroprevalence might be expected. As a possible consequence of our findings, the search for TBEV-positive ticks in these districts may be considered in order to find the exact locations of the new TBE natural foci.

To our knowledge this study shows for the first time a remarkably high prevalence of NT-verified TBEV-specific antibodies in red foxes. In older studies from Germany and the Netherlands, mostly foxes from non-endemic areas were tested and revealed 0% seroprevalence or only one positive sample [23,24]. So far, only one study was performed in a TBE- endemic area in Germany, where the prevalence of antibodies was up to 34.2%; however, the results were not confirmed by NT [14].

Ideally, in epidemiological studies, in each district, the same number of foxes should be sampled. In this case, the determined prevalence can be compared and a meaningful map can be created. The figure in our study is partially misleading, because not all 1233 samples could be represented. The ratio of positive and negative foxes from districts where 10 or more foxes were examined is shown in pie charts. We visualized the localization of the remaining positive foxes by red dots. However, for clarity reasons, the remaining negative foxes (*n* = 315) from districts where less than 10 foxes were sampled are not shown in this figure. The majority of these foxes came from non-risk areas. For detailed data, see Table 1 and Table 2.

The seroprevalence of TBEV-specific antibodies in red foxes from risk areas was found to be significantly associated with the human TBE incidence. This positive correlation demonstrates that foxes are useful to screen for TBE endemicity. Our findings are in line with a study in TBE-endemic areas in Sweden, where the relationship between the red fox population and human TBE incidence was found to be positive [21]. However, this is in contrast to a study in endemic areas of Germany, which described the opposite effect [25].

The relationship between the seroprevalence of foxes from non-risk areas and human TBE incidence was not statistically significant. We found seroprevalences up to 33.3% in districts where the human TBE incidence is 0.0% (City of Gelsenkirchen, Minden-Lübbecke, Nordfriesland) [27]. In none of these districts have human TBE cases been registered so far. There is a need for investigations to see if these discrepancies may be due to low pathogenic TBEV strains that cause human infection with inapparent clinical course. However, since human TBE cases are also known outside of the defined risk areas every year, we hypothesize that such cases are due to TBE foci yet to be discovered. Investigation of fox seroprevalences could help to locate such TBE foci. We conclude that the red fox is a suitable predictive sentinel and can complement the human incidence-based risk assessment.

## 5. Conclusions

Serological monitoring of red foxes appears to be a promising approach to complement the present TBE risk assessment. For epidemiological studies, a two-step diagnostic system with ELISA or IIFA and confirmatory NT is not useful because of the low sensitivity of ELISA and low specificity of IIFA.

## Figures and Tables

**Table 1:** Seroprevalence of TBEV-specific antibodies in red foxes collected in TBE risk areas in Germany. Seroprevalences were only determined for those districts where 10 or more foxes were sampled.

Federal State	District	No. of Sera	No. of Pos.^1^ Sera	Seroprev (%)
Baden-Württemberg	Alb-Donau-Kreis	1	0	n.d.^2^
	City of Baden-Baden	1	1	n.d.
	Biberach/Alb-Donau Kreis	1	0	n.d.
	Böblingen	1	0	n.d.
	Bodenseekreis	17	8	47.1
	Breisgau-Hochschwarzwald-Kreis	21	5	23.8
	Emmendingen	20	11	55.0
	Enzkreis	3	1	n.d.
	Esslingen	3	0	n.d.
	City of Freiburg im Breisgau	5	3	n.d.
	Göppingen	44	14	31.8
	Göppingen/Ostalbkreis	1	0	n.d.
	Heidelberg	2	2	n.d.
	Heilbronn	6	1	n.d.
	Hohenlohekreis	2	1	n.d.
	Karlsruhe	12	3	25.0
	Konstanz	11	5	45.5
	Lörrach	8	3	n.d.
	Ludwigsburg	24	6	25.0
	Main-Tauber-Kreis	3	2	n.d.
	Neckar-Odenwald-Kreis	12	3	25.0
	Ostalbkreis	14	4	28.6
	Rastatt	2	1	n.d.
	Ravensburg	113	41	36.3
	Rems-Murr-Kreis	10	2	20.0
	Reutlingen	3	1	n.d.
	Rhein-Neckar-Kreis	5	2	n.d.
	Rottweil	1	1	n.d.
	Schwäbisch Hall	2	0	n.d.
	Schwarzwald-Baar-Kreis	16	9	56.3
	Sigmaringen	43	21	48.8
	Tübingen	5	0	n.d.
Bavaria	Ansbach	16	5	31.3
	Eichstätt	1	0	n.d.
	Günzburg	11	1	9.1
	Miltenberg	1	1	n.d.
	München	1	0	n.d.
	Neustadt an der Waldnaab	1	0	n.d.
	Roth	3	1	n.d.
	City of Würzburg	1	1	n.d.
Hessen	Bergstraße	1	0	n.d.
	Groß-Gerau	1	1	n.d.
	Offenbach am Main	1	0	n.d.
	Main-Kinzig-Kreis	5	1	n.d.
Lower Saxony	Emsland	18	2	11.1
Saxony	Bautzen	8	1	n.d.
	Erzgebirgskreis	17	1	5.9
	Meißen	33	1	3.0
	Sächsische Schweiz- Osterzgebirge	8	1	n.d.
	Vogtlandkreis	4	1	n.d.
	Zwickau	14	2	14.3
Thuringia	Gera	1	0	n.d.
	Greiz	1	0	n.d.
	Ilm-Kreis	8	2	n.d.
	Saale-Orla-Kreis	1	0	n.d.
**Total**		568	173	30.5

^1^ Pos.: positive; ^2^ n.d.: not detected.

**Table 2:** Seroprevalence of TBEV-specific antibodies in red foxes collected in TBE non-risk areas in Germany. Seroprevalences were only determined for those districts where 10 or more foxes were sampled.

Federal State	District	No. of Sera	No. of Pos.^1^ Sera	Seroprev. (%)
Baden-Württemberg	City of Heilbronn	1	0	n.d.^2^
Brandenburg	Elbe-Elster	1	0	n.d.
	Oder-Spree	2	0	n.d.
	Teltow-Fläming	3	1	n.d.
	Uckermark	3	0	n.d.
Brandenburg/Saxony Anhalt	Havelland/Stendal	2	0	n.d.
			
Hesse	Fulda	9	3	n.d.
	Gießen	2	0	n.d.
	Hersfeld-Rotenburg	1	0	n.d.
	Kassel	2	0	n.d.
	Main-Taunus-Kreis	1	0	n.d.
	Rheingau-Taunus-Kreis	1	0	n.d.
	Waldeck-Frankenberg	3	0	n.d.
	Wetteraukreis	26	2	7.7
Lower Saxony	Cloppenburg	6	2	n.d.
	Cuxhaven	3	1	n.d.
	Delmenhorst	1	0	n.d.
	Diepholz	9	2	n.d.
	Gifhorn	1	0	n.d.
	Göttingen	1	0	n.d.
	Grafschaft-Bentheim	6	2	n.d.
	Hameln-Pyrmont	6	0	n.d.
	Harburg	1	0	n.d.
	Nienburg / Weser	7	2	n.d.
	Osnabrück	21	2	9.5
	Peine	11	2	18.2
	Region Hannover	2	2	n.d.
	Schaumburg	4	1	n.d.
	Stade	5	0	n.d.
	Vechta	8	0	n.d.
	Verden	7	3	n.d.
	Wolfenbüttel	2	0	n.d.
Mecklenburg-Western Pomerania	Ludwigslust-Parchim	2	0	n.d.
	Mecklenburgische Seenplatte	5	0	n.d.
	Rostock	1	0	n.d.
	Vorpommern-Greifswald	10	0	0.0
	Vorpommern-Rügen	2	0	n.d.
North-Rhine Westphalia	Borken	12	4	33.3
	Coesfeld	9	2	n.d.
	Ennepe-Ruhr-Kreis	5	2	n.d.
	Gütersloh	16	2	12.5
	Hamm	1	0	n.d.
	Herford	14	1	7.1
	Hochsauerlandkreis	7	0	n.d.
	Höxter	6	0	n.d.
	Kleve	10	2	20.0
	City of Bielefeld	7	2	n.d.
	City of Düsseldorf	1	0	n.d.
	City of Gelsenkirchen	12	4	33.3
	City of Hagen	2	0	n.d.
	City of Leverkusen	2	0	n.d.
	Lippe	11	0	0.0
	Mettmann	11	0	0.0
	Minden-Lübbecke	15	5	33.3
	Paderborn	2	0	n.d.
	Recklinghausen	1	1	n.d.
	Rhein-Neuss-Kreis	30	2	6.7
	Siegen-Wittgenstein	1	0	n.d.
	Soest	41	2	4.9
	Steinfurt	13	2	15.4
	Unna	4	1	n.d.
	Warendorf	11	2	18.2
	Wesel	9	2	n.d.
Rhineland-Palatinate	Altenkirchen/Westerwaldkreis	2	0	n.d.
	Alzey-Worms	4	1	n.d.
	Bad Dürkheim	5	2	n.d.
	Bitburg-Prüm	1	1	n.d.
	Germersheim	1	0	n.d.
	Kaiserslautern/Kusel	1	0	n.d.
	Mainz-Bingen	3	0	n.d.
	Neuwied	1	1	n.d.
	Rhein-Lahn-Kreis	1	0	n.d.
	Westerwaldkreis	2	0	n.d.
Saxony	Görlitz	36	2	5.6
	City of Leipzig	1	0	n.d.
	Leipzig	15	0	0.0
	Nordsachsen	3	1	n.d.
Saxony-Anhalt	Anhalt-Bitterfeld	8	1	n.d.
	Börde	2	1	n.d.
	Burgenlandkreis	4	0	n.d.
	City of Dessau-Roßlau	5	0	n.d.
	Harz	10	0	0.0
	Jerichower Land	6	0	n.d.
	City of Magdeburg	1	0	n.d.
	Mansfeld-Südharz	3	0	n.d.
	Saalekreis	2	0	n.d.
	Salzlandkreis	11	1	9.1
	Wittenberg	15	1	6.7
Schleswig Holstein	Dithmarschen	3	0	n.d.
	Nordfriesland	12	4	33.3
	Pinneberg	8	1	n.d.
	Plön	1	0	n.d.
	Rendsburg-Eckernförde	9	1	n.d.
	Schleswig-Flensburg	4	1	n.d.
	Segeberg/Steinburg	1	0	n.d.
	Steinburg	1	0	n.d.
	Stormarn	5	0	n.d.
	Stormarn/Herzogtum Lauenburg	6	0	n.d.
Thuringia	Erfurt	6	1	n.d.
	Gotha	6	1	n.d.
	Sömmerda	8	1	n.d.
	Unstrut-Hainich-Kreis	7	3	n.d.
	Weimarer Land	14	1	7.1
**Total**		665	87	13.1

^1^ Pos.: positive; ^2^ n.d.: not detected.

**Table 3:** Results of ELISA in comparison with Neutralization Test (NT) as gold standard, resulting in sensitivity and specificity.

		NT		
		positive	negative	**Total**
**ELISA**	positive	110	11	121
	negative	150	962	1112
	**Total**	260	973	1233
		Sensitivity 42.3%	Specificity 98.9%	

**Table 4:** Intermediate results of 192 samples tested by ELISA in comparison with NT as the gold standard, resulting in sensitivity and specificity.

		NT		
		positive	negative	**Total**
**ELISA**	positive	110	11	121
	negative	13	58	71
	**Total**	123	69	192
		Sensitivity 89.4%	Specificity 84.1%	

**Table 5:** Intermediate results of 192 samples tested by IIFA in comparison with NT as the gold standard, resulting in sensitivity and specificity.

		NT		
		positive	negative	**Total**
**IIFA**	positive	112	26	138
	negative	11	43	54
	**Total**	123	69	192
		Sensitivity 91.1%	Specificity 62.3%	
